# Deciphering nanoparticle protein coronas by capillary isoelectric focusing-mass spectrometry-based top-down proteomics†

**DOI:** 10.1039/d4cc02666g

**Published:** 2024-09-11

**Authors:** Guijie Zhu, Seyed Amirhossein Sadeghi, Morteza Mahmoudi, Liangliang Sun

**Affiliations:** a Department of Chemistry, Michigan State University 578 S Shaw Lane East Lansing Michigan 48824 USA lsun@chemistry.msu.edu; b Department of Radiology and Precision Health Program, Michigan State University East Lansing MI USA

## Abstract

The nanoparticle (NP) protein corona significantly influences the outcome of nanomedicine. We present the first example of top-down proteomics (TDP) measurement of the protein corona using capillary isoelectric focusing-mass spectrometry, identifying seventy proteoforms of 16 cancer-related genes. This technique has the potential to revolutionize our understanding of the protein corona and advance nanomedicine.

## Abstract 2

The first example of top-down proteomics of nanoparticle protein coronas using capillary isoelectric focusing-mass spectrometry.

## Graphical abstract

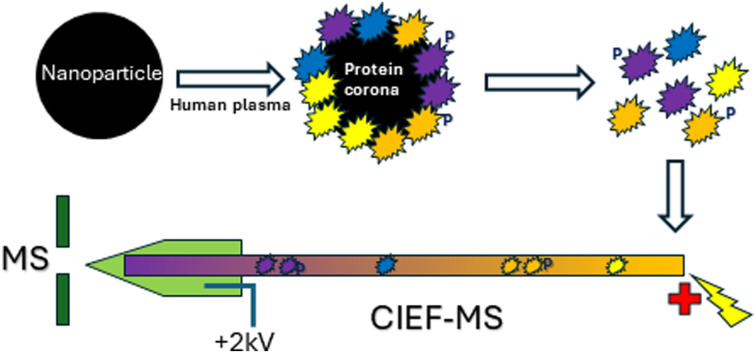

Nanoparticles (NPs) have been increasingly applied in nanomedicine to deliver drugs to specific organs/tissues, to enable tissue imaging, and to carry out disease diagnosis.^1–4^ Once NPs come into contact with biological fluids, *e.g.*, human plasma, their surfaces are covered by a layer of biomolecules (*e.g.*, proteins), called the protein corona.^5^ The composition of the protein corona significantly influences the biological fate of NPs and their therapeutic/diagnostic efficacies.^6–8^ The composition of the protein corona strongly depends on the physicochemical properties of the NPs (*e.g.*, size, shape, and surface functional group).^9–11^ Therefore, a group of NPs with distinct physicochemical properties can be employed to simplify the plasma proteome by capturing a specific pool of proteins in each protein corona, enhancing the depth of detection of low-abundance plasma proteins.^9,11^ Robust and comprehensive characterization of proteins and their proteoforms within the protein corona empowers the nanomedicine community to enhance early disease detection and predict the biological fate of nanomedicine products more accurately.^9^

Bottom-up proteomics (BUP) has been used to offer useful information about gene products in the protein corona.^11,12^ However, BUP fails to determine the exact forms of protein molecules (*i.e.*, proteoforms^13^) in protein coronas due to the enzymatic treatment step and misses valuable protein information, including protein sequence variations (*e.g.*, protein isoforms and truncations) and combinatorial patterns of post-translational modifications (PTMs).^14^ Different proteoforms from the same gene can have substantially different impacts on protein corona and NP interactions with biosystems.^15^ Mass spectrometry (MS)-based top-down proteomics (TDP) directly measures intact proteoforms and is an ideal approach for pursuing a bird's-eye view of the participated proteoforms in protein coronas.^14,16^ High-capacity separations of proteoforms prior to MS are critical for the TDP of complex samples. Capillary electrophoresis (CE)-MS has been well recognized as a useful technique for TDP due to its high-efficiency separation and highly sensitive detection of proteoforms.^16–21^ Capillary isoelectric focusing (cIEF) is one mode of CE and separates proteoforms based on their isoelectric points (pIs) with extremely high resolution.^22,23^ cIEF-MS is an ideal approach for TDP of proteoforms, even protein complexes.^24–28^

In this study, for the first time, an automated cIEF-MS/MS method was developed to measure NP protein corona using TDP, Fig. 1. The protein corona was prepared on polystyrene NPs (PSNPs) according to the used procedure in recent studies.^12,29^ It is noteworthy that we used PSNPs due to our extensive experience in optimizing the parameters involved in the formation of a pure protein corona, ensuring highly accurate and reproducible MS results. Full details on PSNP optimization and characterization for protein corona formation are available in our recent publications.^12,29–31^ Detailed information on protein corona formation is in the Supporting Information I (ESI†). Briefly, PSNPs were incubated with healthy human plasma to form protein coronas. After washing with PBS, the protein corona was eluted from PSNPs using a 0.4% (w/v) SDS solution, followed by buffer exchange to a 100 mM NH_4_HCO_3_ buffer for cIEF-MS/MS.

**Fig. 1:**
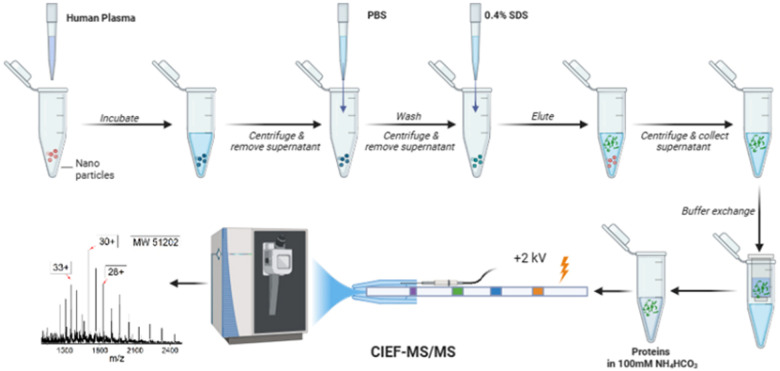
Workflow of cIEF-MS/MS-based TDP for NP protein corona. Polystyrene NPs (PSNPs) were used. The figure was created using BioRender and used here with permission.

We first optimized cIEF-MS/MS regarding ampholyte concentration. Higher ampholyte concentration achieves better separation resolution but also leads to unavoidable ionization suppression of proteoforms. Three concentrations of ampholytes, 1.5%, 1%, and 0.5%, were studied using a standard protein mixture containing cytochrome *c* (cyt *c*, pI 10.8), myoglobulin (Mb, pI 6.9) and carbonic anhydrase (CAs, pI 5.4). Automated cIEF-MS was carried out using the sandwich injection approach,^27,32^ the electrokinetically pumped sheath flow CE-MS interface,^33^ and an Agilent 6545XT Q-TOF mass spectrometer. The three proteins were all baseline separated under the three conditions, Fig. S1 (ESI†). CIEF with a higher concentration of ampholyte could reach a better separation resolution, Table S1 (ESI†). CIEF with a higher ampholyte concentration tends to need a longer analysis time due to the higher buffering capacity of ampholytes, requiring a longer time for titration. Considering the analysis time, separation resolution, and instrument contamination from ampholytes, cIEF-MS with 0.5% ampholytes was employed for the analysis of protein coronas.

Fig. S2 (ESI†) shows the electropherograms of cIEF-MS runs of three protein corona samples (S1, S2, S3) prepared in parallel and each sample was analyzed in technical duplicates. The separation profile and base peak intensity are reasonably consistent across all runs, demonstrating reproducible protein corona analyses. Fig. S3 (ESI†) shows the data of one cIEF-MS run of sample S2. The cIEF-MS observed clear proteoform peaks of large proteins (a, b, and c) and small proteins (d). For example, three and four proteoforms were detected for the 28 kDa (a) and 66 kDa (b) proteins with the relative abundance of those proteoforms resolved. The data demonstrates that cIEF-MS can delineate large and small proteoforms in protein coronas.

To identify proteoforms based on MS/MS, we coupled cIEF to an Orbitrap Exploris 480 mass spectrometer. One protein corona sample was analyzed in technical duplicates by a high-high mode, employing high mass resolution for both MS1 and MS2. The duplicate cIEF-MS/MS runs generated a consistent separation profile and similar numbers of proteoform (63 ± 1, *n* = 2) and protein (25 ± 0, *n* = 2) identifications, Fig. 2A. The identified proteoforms are listed in the Supporting Information II (ESI†). In total, 82 proteoforms and 31 proteins were identified. The two runs shared 43 proteoforms, representing nearly 70% of the number of identified proteoforms in one run, Fig. 2B. The proteoform intensity between the duplicate runs has a clear linear correlation (Pearson's *r* = 0.99), Fig. 2C.

**Fig. 2:**
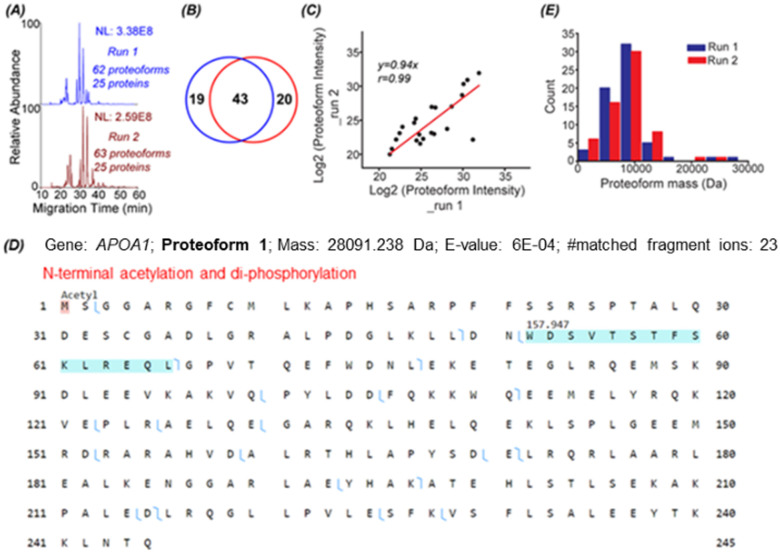
TDP data of protein corona by cIEF-MS/MS using an Orbitrap Exploris 480 mass spectrometer in high-high mode. (A) Base peak electropherograms of duplicate cIEF-MS/MS runs. (B) Venn diagram of proteoform overlaps between duplicate measurements. (C) Proteoform intensity correlation between the duplicate runs. Log 2 (proteoform intensity) was used, and the proteoforms having proteoform feature intensities in both runs were used. (D) Sequence and fragmentation pattern of one APOA1 proteoform (proteoform 1), having one N-terminal acetylation and di-phosphorylation. (E) Mass distribution of proteoforms identified in the two replicate runs.

Three examples of identified proteoforms of gene APOA1 are shown in Fig. 2D and Fig. S4, S5 (ESI†). Proteoform 1 is 28091.238 Da and has one N-terminal acetylation and one 157.947 Da mass shift, Fig. 2D. According to the dbPTM database,^34^ the S and T amino acid residues in this specific amino acid sequence (position 52–66) can be phosphorylated. The deconvoluted MS/MS spectrum of the proteoform shows clear signals of ions corresponding to losses of H_2_O and H_3_PO_4_, Fig. S4 (ESI†). Therefore, the 157.947 Da mass shift should correspond to two phosphorylation events. Proteoform 1 belongs to the level 2A identification.^35^ Proteoform 2 is 22519.954 Da and has N-terminal truncation and a 144.354 Da mass shift between position 195 and 232, Fig. S5 (ESI†). Multiple acetylation (*i.e.*, K) and phosphorylation (*i.e.*, S or T) could happen in this region.^34^ The 144.354 Da may be from the combination of phosphorylation, acetylation, and other PTMs. Proteoform 3 is 18431.319 Da and has N-terminal truncation and one 264.751 Da mass shift, Fig. S6 (ESI†). Proteoforms 2 and 3 are level 3 identifications.^35^ The mass errors of matched fragment ions of the three APOA1 proteoforms are smaller than 10 ppm, and for most fragment ions, especially proteoforms 2 and 3, the mass error is close to 0, Fig. S7 (ESI†). The high mass accuracy of matched fragment ions ensures the high confidence of identifications. The results demonstrate that our cIEF-MS/MS-based TDP could measure diverse proteoforms of the same gene (*i.e.*, APOA1) in the protein corona. Our technique could provide a relative abundance of proteoforms from the same gene. For example, proteoform 1 of gene APOA1 has a substantially higher abundance than others, evidenced by its much higher intensity (2E10 *vs.*<5E6).

APOA1 is a prognostic marker of cancer (https://www.proteinatlas.org/). We identified 12 proteoforms of APOA1. Overall, we identified over 70 proteoforms of 16 cancer-related genes, Table S2 (ESI†). cIEF-MS/MS-based TDP provides an advanced view of the diverse proteoforms in the protein corona, including variations such as truncations and PTMs, as well as their combinations. This proteoform-centric TDP approach has the potential to offer more detailed and accurate information about protein corona composition compared to the traditional peptide-centric BUP. This enhanced accuracy is fundamental for developing and improving safer and more efficient nanomedicines. The data also implies that TDP profiling of protein corona could be useful for discovering novel proteoform biomarkers of diseases, *e.g.*, cancers.

Most of the proteoforms identified in this study using the high-high mode (∼80%) are smaller than 10 kDa, Fig. 2E. The other 20% of the proteoforms are in the mass range of 11–30 kDa. It is challenging for TDP to identify large proteoforms (>30 kDa) from complex samples due to their substantially lower measurement sensitivity compared to small proteoforms.^36^ To improve the measurement quality of large proteoforms, we employed a low-high approach,^37^ utilizing low-resolution MS1 and high-resolution MS2. We detected 24 proteoforms close to or larger than 28 kDa from 4 proteins, Fig. 3 and Fig. S8, and S9 (ESI†). We detected 9 proteoforms from protein 1 (>66 kDa) and 2 proteoforms from protein 4 (>43 kDa), Fig. 3. Based on our capillary zone electrophoresis (CZE)-MS/MS data,^29^ protein 1 should be human serum albumin (HSA). CZE-MS/MS detected three HSA proteoforms and, here, cIEF-MS/MS observed nine HSA proteoforms in a mass range of 66 436–67 625 Da, and the 66 820 Da proteoform is the most abundant one. The theoretical mass of HSA with 17 disulfide bonds (native form) is 66 438 Da. The smallest HSA proteoform detected here (66 436 Da) should be the native form. HSA can be modified by various PTMs, *e.g.*, phosphorylation and glycosylation. The HSA proteoforms detected here must be due to the combinations of PTMs and/or sequence variations. cIEF-MS/MS detected two proteoforms of protein 4 (about 43 kDa), not observed in our CZE-MS/MS study.^29^ For protein 2, cIEF separated it into two peaks (2 and 2′), and each peak has two proteoforms, Fig. S8 (ESI†). Our CZE-MS/MS study only detected the two highly abundant proteoforms of protein 2 (51 200 and 51 860 Da) in one peak.^29^ The nine proteoforms of protein 3 with masses of about 28 kDa (Fig. S9, ESI†) correspond to the products of gene APOA1 based on our high-high mode data, Fig. 2D. The most abundant proteoform of intact APOA1 has an average mass of 28 110 Da, which should be the proteoform in Fig. 2D, having a monoisotopic mass of 28 091 Da (average mass 28 108 Da). The nine proteoforms were separated into three peaks (3, 3′, and 3′′) by cIEF. We only observed five intact APOA1 proteoforms by CZE-MS/MS in one peak.^29^

**Fig. 3:**
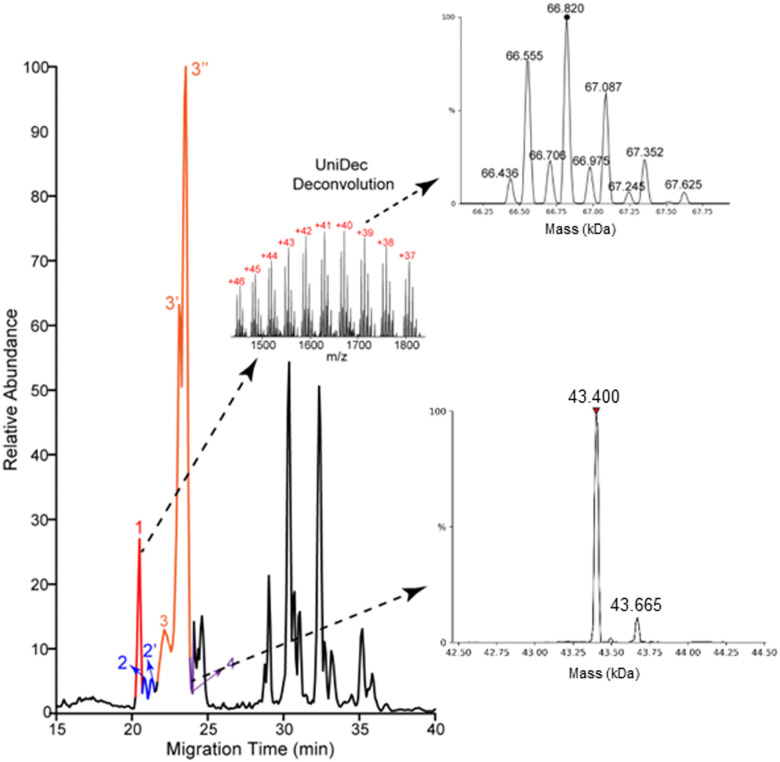
Base peak electropherogram of protein corona by cIEF-MS/MS using an Orbitrap Exploris 480 mass spectrometer in low-high mode. Deconvoluted masses of detected large proteoforms of proteins 1 and 4 are shown. UniDec software^38^ was used for mass deconvolution with default settings.

In summary, our findings demonstrate that cIEF-MS/MS is a superior technique for TDP characterization of protein coronas. It surpasses CZE-MS/MS in large proteoform analysis due to its exceptionally high separation resolution and greater sample loading capacity (400–1000 nL *vs.* 100 nL). This study marks the first investigation of cIEF-MS/MS for TDP of protein coronas. We anticipate that cIEF-MS/MS will significantly advance the field of nanomedicine by providing efficient measurement of small and large proteoforms in protein coronas.

This study has limitations. First, the number of proteoform/gene identifications is much lower than BUP.^29^ We must employ multi-dimensional separations (*e.g.*, LC-cIEF^27^) to boost the proteoform identifications. Second, identifying large proteoforms (>30 kDa) and the accurate localization of PTMs are challenging. The inefficiency of the higher energy collision dissociation (HCD) technique for large proteoform fragmentation is one main reason. We will explore electron-based or photon-based fragmentation methods for better large proteoform identification and PTM localization.^39,40^ We will combine BUP and TDP data for a more robust proteoform characterization.^41^ The low sensitivity of TDP for large proteoforms is another main reason.^36^ Native cIEF-MS could be useful to improve the TDP of large proteoforms in protein coronas because native MS provides much narrower charge state distributions compared to denaturing MS used here.^26^

The authors are thankful for the support from the National Cancer Institute through the grant R01CA247863 (Sun) and the National Institute of Diabetes and Digestive and Kidney Diseases through the grant DK131417 (Mahmoudi). We are thankful for the support from the National Institute of General Medical Sciences through grants R01GM125991 (Sun) and R01GM118470 (Sun), and the National Science Foundation through the grant DBI1846913 (CAREER Award, Sun).

## Conflicts of interest

Morteza Mahmoudi discloses that (i) he is a co-founder and director of the Academic Parity Movement (https://www.paritymovement.org), a non-profit organization dedicated to addressing academic discrimination, violence, and incivility; (ii) he is a co-founder of Targets Tip; and (iii) he receives royalties/honoraria for his published books, plenary lectures, and licensed patents. The authors declare no other competing financial interest.

## Supplementary Material

CC-060-D4CC02666G-s001

CC-060-D4CC02666G-s002

## Data Availability

The proteoform identification data supporting this article has been included as part of the ESI.†
