# Supplementary material for: Deciphering nanoparticle protein coronas by capillary isoelectric focusing-mass spectrometry-based top-down proteomics
Source: Chem Commun (Camb). 2024 Sep 11;60(81):11528–31. doi: 10.1039/d4cc02666g (PMC11418007; doi:10.1039/d4cc02666g)
Supplement: CC-060-D4CC02666G-s002 [file CC-060-D4CC02666G-s002.pdf]

Supplementary Information (SI) for ChemComm.
This journal is © The Royal Society of Chemistry 2024

Supporting Information I

Deciphering nanoparticle protein corona by capillary isoelectric focusing-mass spectrometry-based top-down proteomics

Guijie Zhu,[a] Seyed Amirhossein Sadeghi,[a] Morteza Mahmoudi,[b] Liangliang Sun[a*]

[a] Department of Chemistry, Michigan State University, 578 S Shaw Lane, East Lansing, Michigan 48824, USA. Email: lsun@chemistry.msu.edu

[b] Department of Radiology and Precision Health Program, Michigan State University, East Lansing, MI, USA

Table of Contents

Content	Page
Experimental section	S3-S5
Table S1	S6
Table S2	S7
Figure S1	S8
Figure S2	S9
Figure S3	S10
Figure S4	S11
Figure S5	S12
Figure S6	S13
Figure S7	S14
Figure S8	S15
Figure S9	S16
References	S17

Experimental section

Materials and Chemicals

MS-grade water, methanol (MeOH), formic acid (FA), acetic acid (AA) and fused silica capillaries (50 µm i.d./360 µm o.d., Polymicro Technologies) were purchased from Fisher Scientific (Pittsburgh, PA). Ammonia hydroxide (NH_3H_2O), ammonium acetate (NH_4Ac), ammonium persulfate (APS) Pharmalytes with pI range of 3-10 and 5-8 (GE healthcare), cytochrome c, myoglobin, carbonic anhydrase and 3-(trimethoxysilyl) propyl methacrylate(γ-MAPS) were purchased from Sigma-Aldrich (St. Louis, MO). Acrylamide was purchased from Acros Organics (NJ, USA). A healthy human plasma sample was purchased from Innovative Research (www.innov-research.com) and diluted to 55% using phosphate buffer solution (PBS, 1X). Poly Styrene Nanoparticles (PSNPs, ~100 nm) were bought from Polysciences (www.polysciences.com).

Sample preparation

A mixture of standard proteins with cytochrome c (0.1 mg/mL), myoglobin (0.1 mg/mL), carbonic anhydrase (0.5 mg/mL) was prepared in 10 mM NH_4AC.

For protein corona sample preparation, three batches of samples were prepared in parallel. In detail, 75 µL PSNPs (25 mg/mL) were incubated with 55% human plasma (1 mL) for 1 h at 37 °C with constant shaking. Then, the mixture was centrifugated at 14,000xg for 20 min to remove the supernatant. Then the protein coronas were washed by cold PBS twice. For protein elution, the protein-NP complexes were incubated in a 0.4% (w/v) SDS solution for 1.5 hours at 60°C with constant shaking, The mixture was centrifuged at 19,000xg for 20 minutes at 4°C and the supernatant was collected for buffer exchange step. An Amicon Ultra Centrifugal Filter with a Molecular Weight Cut-Off (MWCO) of 10 kDa was employed. First, the filter was wet by using 200 µL of 100 mM ammonium bicarbonate (pH 8.0), followed by centrifugation at 14,000xg for 10 minutes. Then, 200 µg of proteins were loaded to the filter, and centrifugation was performed at 14,000xg for 20 minutes. After that, 200 µL of 8 M urea in 100 mM ammonium bicarbonate solution was added, followed by centrifugation at 14,000xg for 20 minutes. This step was repeated twice. Finally, three additional rounds of buffer exchange by using 100 mM ammonium bicarbonate were done to generate the sample with the final volume around 50 µL.

After buffer exchange, the concentration of total proteins was determined using a bicinchoninic acid (BCA) kit (Fisher Scientific) in accordance with the manufacturer's instructions. The final protein concentration of three batches of sample (S1, S2, S3) in 100 mM ammonium bicarbonate is around 1.5 mg/mL.

Preparation of linear polyacrylamide (LPA)-coated capillary

The LPA-coating procedure is based on references 1 and 2. The capillary was flushed with 1 M hydrochloric acid, water, 1 M sodium hydroxide, water, and finally washed with

methanol for 50 times of volume of capillary by using a hand pump. The capillary was then dried under a flow of nitrogen at room temperature for 4 h. Next, 50% (v/v) 3-(trimethoxysilyl) propyl methacrylate in methanol was flushed through the capillary for 10 min. Both ends of the capillary were sealed, and the filled capillary was incubated at room temperature for 24 h. The capillary was then rinsed with methanol for 10 min and dried under nitrogen. Forty milligrams of acrylamide were dissolved in 1 mL water, and 2 μL of 5% (w/v) ammonium persulfate was added to 500 μL of the acrylamide solution. The mixture was vortexed for 30 s and degassed for 5 min using nitrogen. Then, the mixture was introduced into pretreated capillary by vacuum. With both ends sealed, the capillary was incubated in a 50°C water bath for 60 min. The capillary was then flushed with water to remove excess reagents. An approximately 5 mm length of the outside of the distal tip of the capillary was etched with HF for 75 min to reduce the outer diameter of the etched part to ~70 μm; the detailed protocol for HF etching is described in reference 3.

Capillary isoelectric focusing (CIEF)-MS analysis

A 100-cm LPA-coated capillary (50 μm i.d.) was used for CIEF separation of standard proteins for investigation of ampholytes concentration and an 80 cm long LPA-coated capillary (50 μm i.d.) was used for CIEF separation of protein corona sample. The ampholyte solution is a mixture of pI 3-10 and 5-8 ampholytes with a volume ratio of 1:2. An autosampler was used for automated sample injection and high voltage application. The sandwich injection CIEF strategy was applied as we published before. [4,5] For corona sample analysis, a segment of 8 cm catholyte (0.3% NH_3H_2O) was injected using pressure injection followed by a segment of 20 cm sample and ampholytes mixture using pressure injection. For the standard protein experiment, a segment of 8 cm catholyte (0.3% NH_3H_2O) was injected using pressure injection followed by a segment of 50 cm sample and ampholytes mixture. Then a voltage of 30kV was applied to perform protein focusing and mobilization steps. EMASS-II (CMP Scientific) interface was used to couple the separation to the mass spectrometer. [6,7] A 2.0 kV spray voltage was applied on the ~30 μm glass spray emitter containing 0.2% (v/v) formic acid and 10% (v/v) methanol sheath liquid. An Agilent 7100 CE System with Agilent 6545XT Q-TOF mass spectrometer was used for ampholyte investigation experiments and initial investigations of protein corona samples. A CESI 8000 Plus (Beckman Coulter) coupled to an Orbitrap Exploris 480 mass spectrometer (Thermo Fisher Scientific) was used for protein corona identification experiments.

Agilent 6545XT Q-TOF mass spectrometer Full scan mass spectra were collected in the m/z range of 600-2000 with the acquisition rate of 0.5 spectra per s. Orbitrap Exploris 480 mass spectrometer in Data-dependent acquisition (DDA) mode was employed. *For the high-high approach*, the Full MS parameters included a high mass resolution of 480,000 (at m/z 200) with a single microscan, covering a scan range of 600-2000 m/z. Precursor ions in Full MS spectra were isolated with a 2 m/z window and subjected to fragmentation through higher-energy collisional dissociation (HCD) with a normalized collision energy

(NCE) of 25%. Only precursor ions with an intensity exceeding 1E4 and a charge state ranging from 5 to 60 were selected for fragmentation. Product ions were detected with a resolution of 120,000 (at 200 m/z), utilizing 3 microscans, and maintaining a normalized AGC target value of 100% for both conditions. Dynamic exclusion was enabled with a duration of 30 seconds and a mass tolerance of 10 ppm (parts per million). Additionally, the "Exclude isotopes" function was activated. *For the low-high approach*, the MS1 resolution was 7500 (at m/z 200) with 10 microscans. Other parameters are the same as the high-high approach.

Data analysis

Proteoform identification was performed on a home-built protein database (~1,000 protein sequences), in which the proteins identified in the bottom-up proteomics data were included. [8] The TopPIC (Top-down mass spectrometry-based Proteoform Identification and Characterization) pipeline was used for the database search of *the "high-high" approach* data for proteoform identification. [9] RAW files were first converted into mzML files using the Msconvert tool. [10] Then, the spectral deconvolution was done using TopFD (Top-down mass spectrometry Feature Detection, version 1.6.2). [11] The database search was performed using TopPIC (version 1.6.2). [9] The maximum number of unexpected mass shifts was one. The mass error tolerances for precursors and fragments were 10 parts per million (ppm). A maximum mass shift of 500 Da for the unknown mass shifts was chosen. A 1% spectrum-level and 1% proteoform-level false discovery rates (FDRs) based on the target-decoy approach were used to filter the proteoform-spectrum matches (PrSMs) and proteoform identifications. The TopDiff (Top-down mass spectrometry-based identification of Differentially expressed proteoforms, version 1.6.2) software was used to perform label-free quantification of identified proteoforms using default settings. [12]

Table S1. Separation resolution of proteins after analyzed by cIEF-MS.

	Resolution of cyt c and Mb	Resolution of Mb and CAs
1.5% Ampholytes	5.4	3.6
1% Ampholytes	4.4	3.2
0.5 % Ampholytes	4.2	2.6

Table S2. Summary of identified proteoforms of cancer-related genes. *

	Gene	Protein Name	Prognostic summary	Proteoforms identified
1.	SAA1	Serum amyloid A-1	Prognostic marker in renal cancer (unfavorable)	7
2.	APOA1	Apolipoprotein A-I	Prognostic marker in renal cancer (unfavorable) and liver cancer (favorable)	12
3.	TTR	Transthyretin	Cancer-related genes<br>FDA approved drug targets	1
4.	APOC1	Apolipoprotein C-I	Prognostic marker in liver cancer (favorable)	1
5.	APOA2	Apolipoprotein A-II	Cancer enriched (liver cancer)	18
6.	APOC2	Apolipoprotein C-II	Cancer enriched (liver cancer)	13
7.	APOC4-APOC2	Apolipoprotein C-II	Cancer enriched (liver cancer)	1
8.	APOC3	Apolipoprotein C-III	Prognostic marker in renal cancer (unfavorable) and liver cancer (favorable)	5
9.	CLU	Clusterin	Prognostic marker in thyroid cancer (favorable)	3
10.	C3	Complement C3	Prognostic marker in renal cancer (unfavorable) and liver cancer (favorable)	4
11.	APOE	Apolipoprotein E	Cancer enhanced (liver cancer)	2
12.	ITIH1	Inter-alpha-trypsin inhibitor heavy chain H1	Prognostic marker in liver cancer (favorable)	1
13.	ITIH4	Inter-alpha-trypsin inhibitor heavy chain 4	Cancer enhanced (liver cancer)	2
14.	FGA	Fibrinogen alpha chain	Prognostic marker in renal cancer (unfavorable) and liver cancer (favorable)	1

15.	APOB	Apolipoprotein B-100	Cancer enhanced (liver cancer)	1
16.	C4A	Complement C4-A	Cancer enhanced (liver cancer)	1

* The prognostic summary is from the Human Protein Atlas (https://www.proteinatlas.org/).

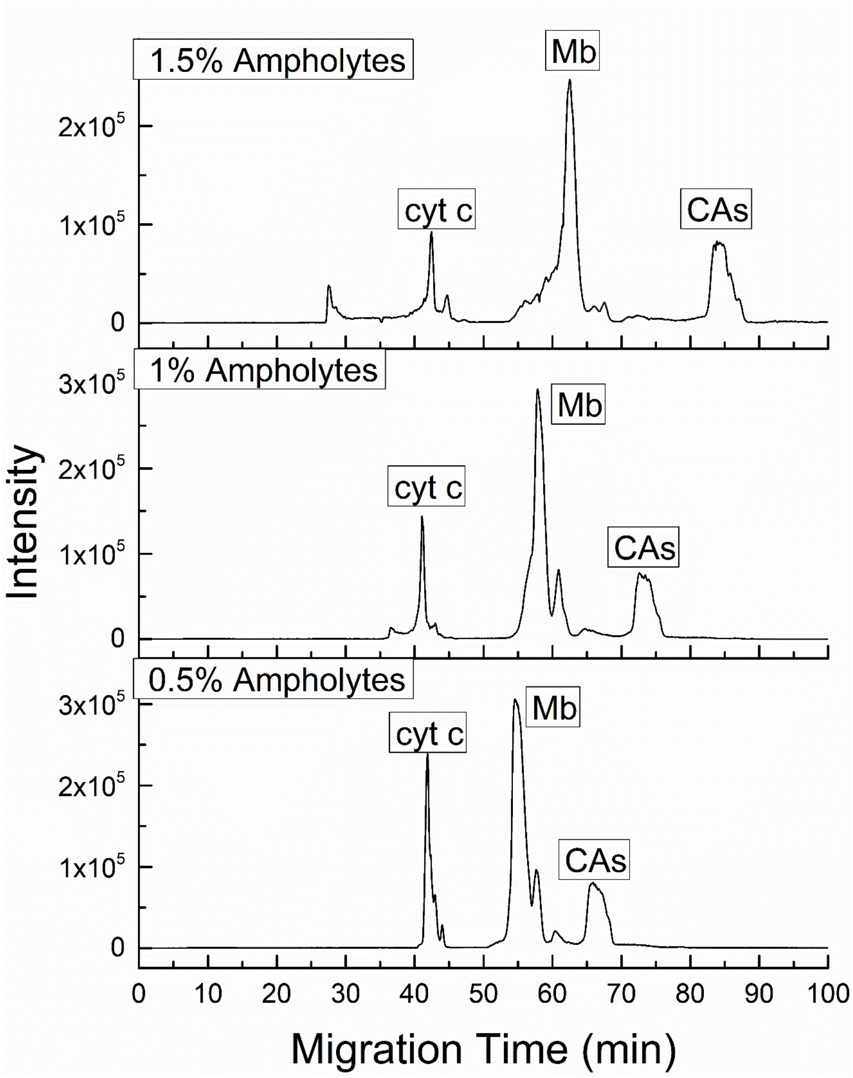

Figure S1. cIEF-MS analysis of a standard protein mixture (cytochrome c (cyt c), myoglobin (Mb), and carbonic anhydrase(CAs)) with three different ampholyte concentrations (1.5%, 1%, and 0.5%). An Agilent 6545XT Q-TOF mass spectrometer was used. A 1-meter-long LPA-coated capillary was used for separation.

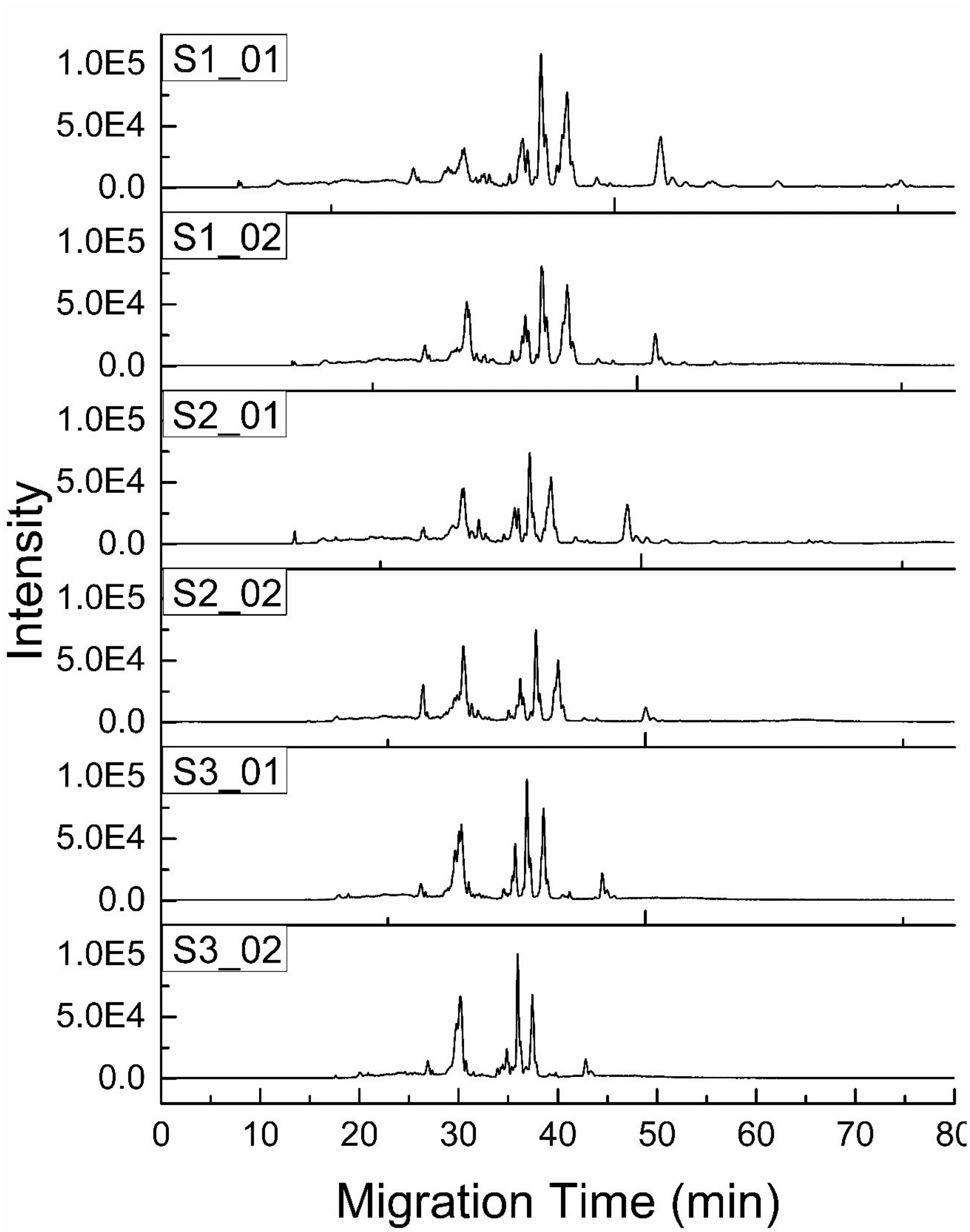

Figure S2. Electropherograms of cIEF-MS analysis of three protein corona samples prepared in parallel (S1, S2, S3). Each sample was measured in duplicate cIEF-MS runs (S1_01 and S1_02, S2_01 and S2_02, S3_01 and S3_02). An Agilent 6545XT Q-TOF mass spectrometer was used. An 80-cm-long LPA-coated capillary was used for separation.

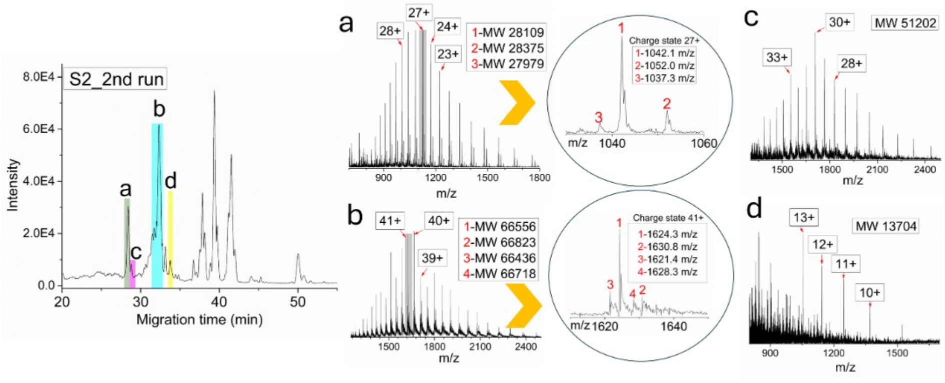

Figure S3. An example electropherogram of cIEF-MS analysis of protein corona sample 2 and mass spectra of four example proteins (a, b, c, and d). The deconvoluted masses of proteoforms of those proteins are labelled. Zoom-in mass spectra of the charge state +27 of protein a and +41 of protein b are shown. The mass deconvolution was performed using the ESIprot [13] online tool using default settings.

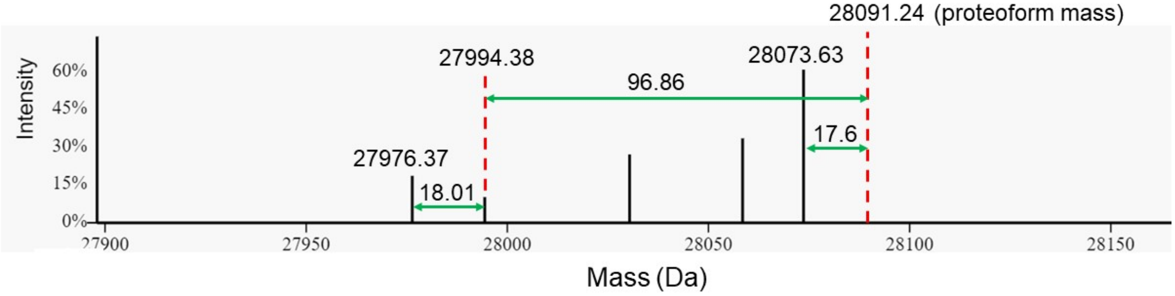

Figure S4. The deconvoluted mass spectrum of **Proteoform 1** of *APOA1* in Figure 2D. Only the high mass region is shown here. The 17.6 and 18.01 Da should represent H_2O loss. The slight mass discrepancy between 17.6 Da and the theoretical mass of H2O (18.01 Da) may be due to the mass error of the measurement for those large ions. The 96.86 Da should represent the H_3PO_4 loss. The about 1-Da mass difference between the 96.86 Da and the theoretical mass of H_3PO_4 (97.99 Da) may be due to the monoisotopic peak assignment error during the mass deconvolution.

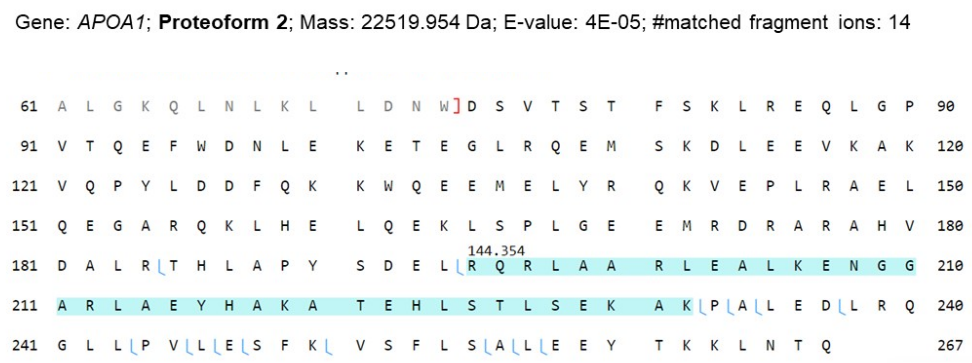

Figure S5. Sequence and fragmentation pattern of one *APOA1* proteoform (Proteoform 2), which has one N-terminal truncation and one 144.354-Da mass shift.

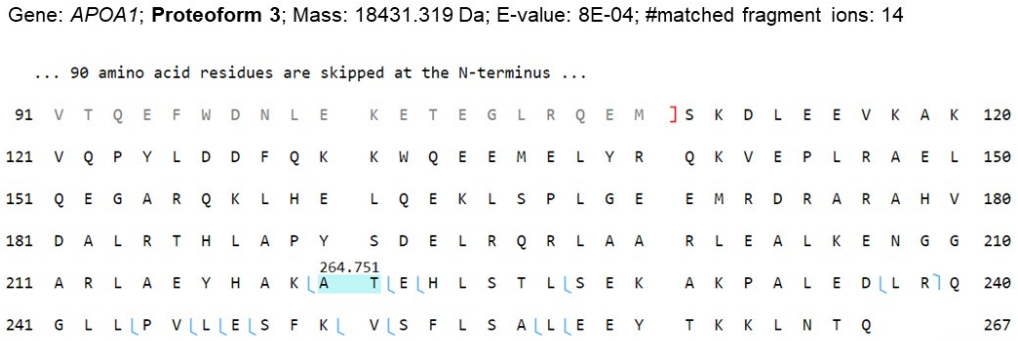

Figure S6. Sequence and fragmentation pattern of one *APOA1* proteoform (Proteoform 3), which has one N-terminal truncation and one 264.751-Da mass shift.

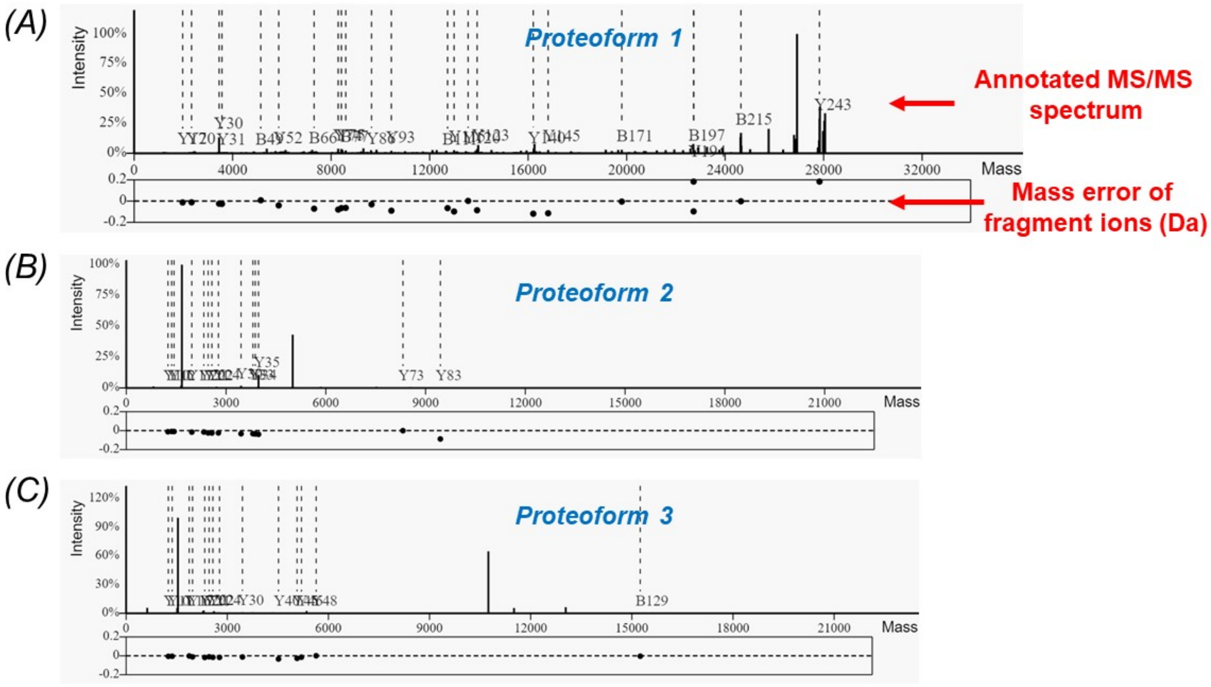

Figure S7. The annotated MS/MS spectra and mass errors of matched fragment ions of the three proteoforms of *APOA1*. (A) Proteoform 1; (B) Proteoform 2; (C) Proteoform 3.

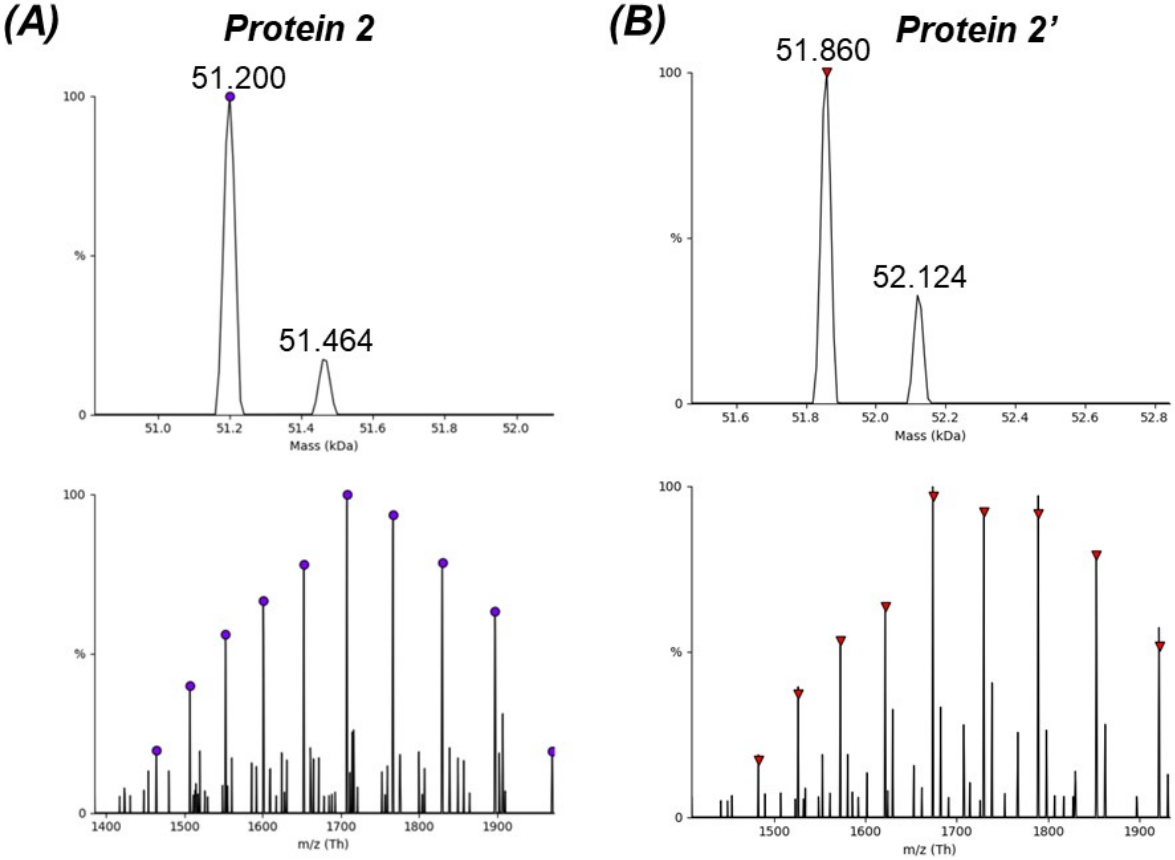

Figure S8. Mass spectrum and deconvoluted masses of Protein 2 (A) and Protein 2' (B). Protein 2 and 2' are based on Figure 3. UniDec software [14] was used for mass deconvolution with default settings.

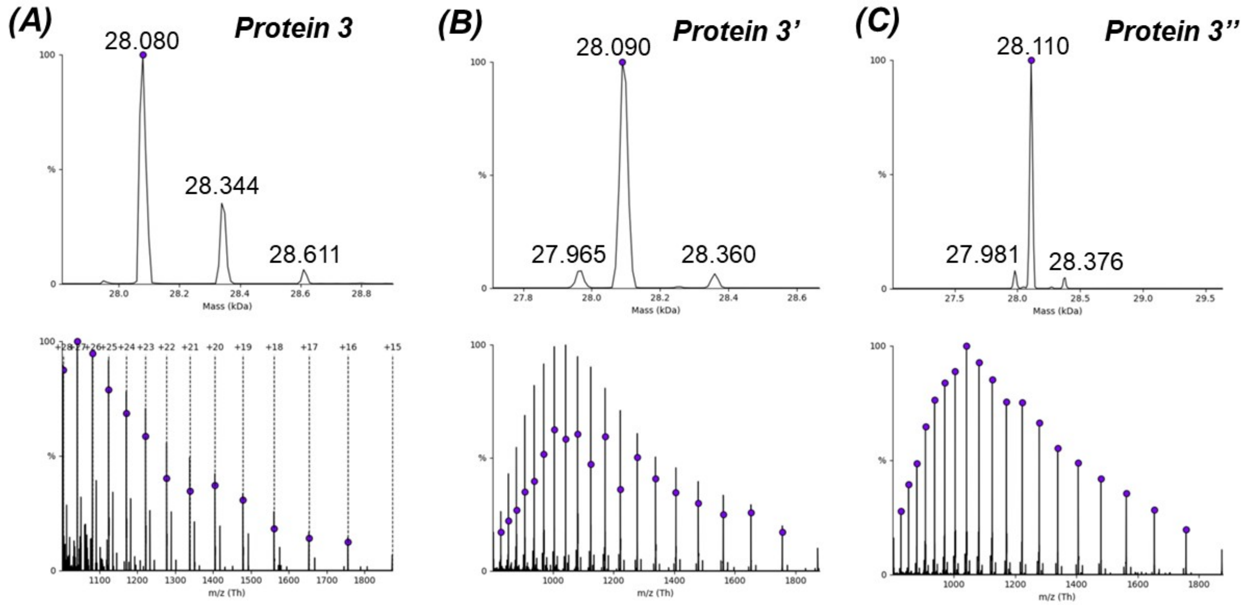

Figure S9. Mass spectrum and deconvoluted masses of Protein 3 (A), Protein 3’ (B), and Protein 3’’ (C). Protein 3, 3’, and 3’’ are based on Figure 3. UniDec software [14] was used for mass deconvolution with default settings.

References

1. Zhu G, Sun L, Dovichi NJ. Talanta. 2016 Jan 1;146:839-43.

2. Chen D, Shen X, Sun L. Analyst. 2017 Jun 21;142(12):2118-2127.

3. Sun L, Zhu G, Zhao Y, Yan X, Mou S, Dovichi NJ. Angew Chem Int Ed Engl. 2013 Dec 16;52(51):13661-4.

4. Zhu G, Sun L, Dovichi NJ. J Sep Sci. 2017 Feb;40(4):948-953.

5. Xu T, Shen X, Yang Z, Chen D, Lubeckyj RA, McCool EN, Sun L. Anal Chem. 2020 Dec 15;92(24):15890-15898.

6. Sun L, Zhu G, Zhang Z, Mou S, Dovichi NJ. J Proteome Res. 2015 May 1;14(5):2312-21.

7. Wojcik R, Dada OO, Sadilek M, Dovichi NJ. Rapid Commun Mass Spectrom. 2010 Sep 15;24(17):2554-60.

8. Sadeghi SA, Ashkarran AA, Mahmoudi M, Sun L,

doi: https://doi.org/10.1101/2024.03.22.586273.

9. Kou Q, Xun L, Liu X. Bioinformatics. 2016 Nov 15;32(22):3495-3497.

10. Kessner D, Chambers M, Burke R, Agus D, Mallick P. Bioinformatics. 2008, 24, 2534– 2536.

11. Rehman Basharat A, Zang Y, Sun L, Liu X. Anal Chem. 2023 May 30;95(21):8189-8196.

12. Lubeckyj RA, Rehman Basharat A, Shen X, Liu X, Sun L. J Am Soc Mass Spectrom. 2019 Aug;30(8):1435-1445.

13. Winkler R. Rapid Commun. Mass Spectrom., 2010, 24, 285-94.

14. Marty MT, Baldwin AJ, Marklund EG, Hochberg GKA, Benesch JLP, Robinson CV. Anal Chem., 2015, 87, 4370-6.
